# Postnatal care service utilisation for babies within the first two months after childbirth: an analysis of rural-urban differences in eleven Sub-Saharan African countries

**DOI:** 10.1186/s12884-023-05758-4

**Published:** 2023-06-07

**Authors:** Kwamena Sekyi Dickson, Castro Ayebeng, Addae Boateng Adu-Gyamfi, Joshua Okyere

**Affiliations:** 1 Department of Population and Health, University of Cape Coast, Cape Coast, Ghana; 2 Department of Nursing, College of Health Sciences, Kwame Nkrumah University of Science and Technology, Kumasi, Ghana

**Keywords:** Postnatal care services, Utilisation, Sub-Saharan Africa

## Abstract

**Background:**

The World Health Organisation recommends that all mothers seek postnatal care (PNC) within the first two months after childbirth. This study examined PNC utilisation for babies within the first two months after childbirth.

**Methods:**

We used data from the most recent Demographic and Health Surveys (DHS) (2018–2020) of eleven countries in SSA. Descriptive and a multivariate analysis were carried out, and presented in adjusted odds ratios. The explanatory variables included: age, place of residence, level of formal education, wealth quintile, antenatal care visits, marital status, frequency of watching TV, listening to radio and reading newspaper, getting permission to go medical help for self, getting money needed for treatment, and distance to facility.

**Results:**

PNC utilisation was 37.5% and 33% in urban and rural residences, respectively. Higher level of education (Urban: AOR = 1.39, CI = 1.25, 1.56; Rural: AOR = 1.31, CI = 1.10, 1.58), 4 or more ANC visits (Urban: AOR = 1.32, CI = 1.23, 1.40; Rural: AOR = 1.49, CI = 1.43, 1.56 0.86), requiring permission to go to the health facility (Urban: AOR = 0.67, CI = 0.61, 0.74; Rural: AOR = 0.86, CI = 0.81, 0.91), listening to the radio at least once a week (Urban: AOR = 1.32, CI = 1.23, 1.41; Rural: AOR = 0.86, CI = 0.77, 0.95), and watching television at least once a week (Urban: AOR = 1.11, CI = 1.03, 1.21; Rural: AOR = 1.15, CI = 1.07, 1.24) were significantly associated with PNC service utilisation in both rural and urban areas. However, belonging to a richer wealth status (AOR = 1.11, CI = 1.02, 1.20) and having a problem with distance (AOR = 1.13, CI = 1.07, 1.18) were significant in only rural areas, while having a problem with money for treatment was significant only in urban areas (AOR = 1.15, CI = 1.08, 1.23).

**Conclusion:**

In this study, we conclude that the PNC service utilisation within the first 2 months after delivery was low across rural and urban residences. There is, therefore, a need for SSA countries to develop population tailored interventions such as advocacy and health education targeted at women with no formal education in both rural and urban areas. Our study also suggests that SSA countries must intensify radio programs and advertisements on the health benefits of PNC to improve maternal and child health.

**Supplementary Information:**

The online version contains supplementary material available at 10.1186/s12884-023-05758-4.

## Background

The postnatal period (i.e., the first 6–8 weeks after birth) is regarded as critical for the survival of both mothers and their newborn babies [1]. Available evidence shows that more than 60% of the world’s maternal mortality occurs within the postpartum period, with a substantial proportion of these deaths occurring within the first two months after delivery [1]. In 2020, there were 2.4 million newborn deaths that occurred within the postnatal period [2]. Specifically, Sub-Saharan Africa (SSA) and South Asia contribute significantly to maternal mortality within the postnatal period [3]. For instance, a study conducted in South Africa revealed that nearly 76% of maternal deaths were recorded within the early phase (i.e., first two days) of the postnatal period [4]. The World Health Organisation (WHO) also reports that 74.3% of neonatal deaths occurred within the first week of life [1]. Thus, emphasising the need to prioritise PNC services, particularly in SSA. The relevance of PNC to combating maternal mortality has been recognised at the highest level of international development. As such, there is the ratified sustainable development goal (SDG) 3 target 2 which aims to reduce neonatal mortality to 12 per 1,000 live births, and under-5 mortalities to 25 per 1,000 live births. The SDG further envisions to provide PNC services to 90% of mothers worldwide by the year 2030 [5].

PNC services that are provided within first two days (i.e., 48 h) after childbirth is referred to as early PNC [6]. The World Health Organisation (WHO) recommends that the first PNC begins within 24 h after childbirth, then the second PNC service provided on the third day; the third and last PNC are recommended to take place between days 7 and 14, and before the end of the sixth week respectively [1, 7]. Previous studies have established that PNC utilisation is essential in planning the health needs of the mother and the newborn child [8, 9]. During PNC sessions, service providers discuss with mothers the importance of exclusive breastfeeding for at least six months, the proper dietary and nutritional needs of the child as well as the need to sleep under insecticide bed nets to prevent mosquito bites [7]. Moreover, it is during PNC sessions that the child’s birth register is complemented, while mothers are encouraged to ensure that the child receives a full complement of immunization [10, 11]. The WHO further recommends that all mothers seek PNC within the first two days after childbirth because, the first week of life is critical to the survival of the newborn [1].

Notwithstanding, there seems to be heterogeneity in the utilisation of PNC in Sub-Saharan Africa. In Uganda, 50% of mothers utilise PNC [6]; however, a different study from South Sudan revealed that only 11.4% of mothers utilised PNC [12]. The existing studies conducted in individual countries indicate that some of the determinants of early PNC utilisation include place of residence, employment status, maternal level of education, ANC attendance and access to health facilities [6, 7, 12]. For instance, Appiah et al. [7] reported that women who were employed as well as those who considered distance to health facilities as unproblematic were more likely to utilise PNC services. The heterogeneity in the findings of the various studies could be due to the differences in defining PNC. While Appiah et al. [7] referred to PNC utilisation as starting ‘immediately after childbirth and goes into 6 weeks (42 days) after childbirth’, Ndugga et al. [6] defined it as ‘having received a postnatal checkup from a skilled health provider within 2 days after childbirth.’

Nevertheless, there is a paucity of empirical studies based on nationally representative datasets in SSA to explore the nuances of PNC utilisation within the first two months after delivery. The extant studies are country-specific, and as such, they do not reflect the overall picture in SSA. There are, however, two studies in SSA that used demographic and health survey data to investigate PNC [13, 14]. Nonetheless, Benova et al.’s study did not focus on PNC within the first two months but rather focused on women receiving a postpartum health check by a health professional before discharge from a health facility following childbirth [13]. In Tessema et al.’s study [14], the time period was limited to only first two days; moreover, the authors did not disaggregate the findings by place of residence (rural-urban areas). Realizing the importance of rural-urban disparities in the context of PNC utilisation is worth studying. A substantial body of research demonstrates the critical role of rural-urban disparities and access to health facilities in improving care utilisation and health outcomes [15–18]. Access to care in urban settings is commonly assumed to be advantageous to that of rural residents; people living in rural areas are more likely to travel long distances for care, especially secondary and tertiary care [14]. Also, rural dwelling women have limited options for healthcare facilities [14].

Recognizing variations within rural and urban disparities has important implications for achieving Universal Health Coverage (UHC) and, eventually, meeting global maternal and infant death reduction targets, since access to essential PNC services can enhance maternal and child health. The DHS uses country-specific definitions for urban-rural divide which is usually based on population thresholds, population density, or the presence of infrastructure. This gap in literature makes it difficult to understand the dynamics of PNC utilisation within the first two months after delivery from a regional perspective. We, therefore, sought to narrow this literature gap by examining This study examined PNC utilisation for babies within the first two months after childbirth using the most recent data from eleven SSA countries. Moreover, unlike previous studies that have focused solely on rural settings [7] or generally analysed mothers’ utilisation of PNC services [6], we disaggregate our findings by place of residence. Hence, allowing us to ascertain which determinants significantly predicted PNC utilisation only in the rural areas, determinants that were only significant in the urban areas and those that were across both places of residence.

## Methods

### Data source

We used data from the most recent Demographic and Health Surveys (DHS) conducted between 2018 and 2020 in eleven countries: Benin (2018), Cameroon (2018), Gambia (2020), Guinea (2018), Liberia (2020), Mali (2018), Nigeria (2018), Rwanda (2020), Sierra Leone (2019), Senegal (2019), Zambia (2018) in SSA. The surveys selected nationally representative samples of women in their reproductive age (15–49 years) groups using a two-stage stratified cluster sampling approach. Because it collects precise data on maternal (antenatal care, delivery, and postnatal care), child health, fertility, family planning, infant and child mortality, and other areas, the DHS is ideal for our study. Women who had given birth up to two years before to the survey were included in the study. Data on PNC was captured on women who had given birth in the 2 years preceding the survey. The study included a total of 82,214 (rural-53,758; urban-28,456) women from the eleven countries. Following a review of our concept note, the MEASURE DHS granted permission to use the data set. The datasets are available to the public for free at The DHS Program - Available Datasets.

### Study variables and measurements

#### Outcome variable

The outcome variable employed for this study was Baby Postnatal care service utilisation within 2 months after delivery. Postnatal utilisation was derived from the question “did baby received Postnatal care service within 2 months after delivery ?” The response was recoded as yes (coded as 1) and no (coded as 0).

#### Explanatory variables

Eleven explanatory variables were used in accordance with both theoretical and empirical literature [19–21]. The main explanatory variables include maternal age (15–19, 20–24, 25–29, 30–34, 35–39, 40–44, 45–49 years), place of residence (urban, rural), level of formal education (no education, primary, secondary, higher) wealth quintile (poorest, poorer, middle, richer, richest), Antenatal care (ANC) visits (less than 4, 4 or more), marital status (never in union, married, cohabitation, widowed, divorced, separated), frequency of listening to radio (not at all, less than once a week, at least once a week), frequency of watching television (not at all, less than once a week, at least once a week)- Radio and television were assumed important in the analysis due to the possibility of radio/television advertisement and education programmes on health benefits of PNC. Also, getting permission to go get medical help for self (Big problem, Not a big problem), getting money needed for treatment (Big problem, Not a big problem), and distance to facility (Big problem, Not a big problem) are some reported serious problems in accessing health care which have implications on PNC utilisation within the first two months.

The DHS program defines an urban area by certain characteristics. Areas that fell out of these characterizations were considered to be rural:

1. Every city or municipality having a population density of at least 1,000 persons per square kilometer.
2. Each central district of municipality or city which has a population density of at least 500 persons per square kilometer.
3. Each central district (not included in 1 and 2), regardless of the population size which has the following:

- Street pattern, i.e., network of streets in either parallel or right-angle orientation.
- At least six establishments (commercial, manufacturing, recreational, and/or personal services).
- At least three of the following: (i) A town hall, church, or chapel with religious services at least once a month; (ii) A public plaza, park, or cemetery; (iii) A market place or building where trading activities are carried on at least once a week; (iv) A public building like a school, hospital, puericulture and health center or library.

#### Inclusion and exclusion criteria

The study included women with live birth experience in the two years preceding the survey. Women with stillbirths were excluded since the focus of this study was on the baby’s access to PNC services. Notwithstanding, a sample size of 82,214 was large enough with high statistical power to generate robust estimates. To address any potential sampling bias arising from under or over-sampling of participants, we employed sample weighting techniques. Specifically, we utilized the individual sampling weight variable, v005, from the dataset of the women’s file. By applying these sample weights, we accounted for the complex survey design and ensured that our estimates were representative of the total population. Regarding clustering, we acknowledge the hierarchical nature of the DHS dataset, where respondents are nested within survey clusters. To mitigate the potential bias in standard errors caused by this clustering effect, we adopted the Huber-White technique. This approach allowed us to derive robust standard errors, which provide more accurate estimates by accounting for the hierarchical structure of the data [22].

#### Analytical procedure

Descriptive analysis was carried out to compare the prevalence of PNC within the first two months in the rural and urban settings of the selected countries. Chi-square test was used to examine the association between PNC and the explanatory variables. Multivariate analysis was then utilised to determine the links between the outcome and the respondents’ explanatory variables, using a logistic regression model [23]. There were two models made. The relationship between the independent variables and the outcome variable among urban residence was investigated in Model 1. Model 2 looked at the relationship between the independent variables and the outcome variable among rural residence. A multicollinearity test was performed on each variable in the models. The odds ratios for each variable were calculated using a 95% confidence range. Stata was used to handle and analyse the data (Version 17). All methods were carried out in accordance with relevant guidelines (i.e., the Strengthening the reporting of observational studies in epidemiology [STROBE] statement] and the Declaration of Helsinki).

### Results

### Descriptive analysis

#### Background characteristics

Generally, the prevalence of postnatal care service utilisation within the first 2 months after delivery was 37.5% in urban and 33% in rural residence. The prevalence of PNC utilisation within the first 2 months after delivery in urban residence ranged from 16.3% in Rwanda to 94.0% in Senegal. Whereas the prevalence of PNC utilisation within the first 2 months after delivery in the rural residence ranged from 16.3% in Nigeria to 81.5% in Senegal (see Table 1). There were four out of the 11 countries where PNC utilisation was higher in rural areas than in the urban areas: Gambia, Liberia, Rwanda and Sierra Leone.

For women living in urban areas, 38.3% of those aged 30–34 utilised PNC services. Similarly, for women with richest wealth status, with 4 or more ANC visits, and married, 37.7%, 39.3% and 38.9% respectively utilised PNC services. Concerning level of education, 38.9% of those with higher education used PNC within the first 2 months after delivery. The proportion of PNC utilisation within the first 2 months was high among women who listen to the radio (66.7%) and watch television (68.6%) compared to those who did not listen to the radio or watch TV. Women who had a big problem getting permission to go for medical help (39.1%), big problem with getting money needed for treatment (39.1%), and big problem with distance, (38.4%) had lower utilisation of PNC services compared to those who did not have a big problem with three accessibility indicators (see Table 2).

Comparatively, among women residing in the rural areas, 33% of those aged 30–34 utilised PNC within the first 2 months. Regarding level of education, 34.6% of women with higher education utilised PNC. Among women with richest wealth status, those with 4 or more ANC visits, and married women, 31.7%, 39.8% and 33.6% utilised PNC services, respectively. Also, the proportion of PNC utilisation was higher among those radio (69%) and those who watched television almost every day (64.8%) compared to those who did not listen to the radio or watch TV at all. Relatedly, 34.3%, 35.3%, and 31.9% of women who had a big problem getting permission to go for medical help for self, getting money needed for treatment, and with distance respectively utilised PNC services within 2 months after delivery (see Table 2).

**Table 1:** Country, survey year and postnatal care service utilisation within the first 2 months after delivery

Variable	Urban		Rural	
	(n)	Baby Postnatal care service utilisation within 2 months after delivery (%)	(n)	Baby Postnatal care service utilisation within 2 months after delivery (%)
Country	X^2^ = 864.0; p < 0.001		X^2^ = 1.7; p < 0.001	
Benin 2018	3,514	23.5	5,226	17.6
Cameroon 2018	2,980	30.0	3,390	24.7
Gambia 2020	2,679	52.8	3,046	69.8
Guinea 2018	1,561	38.2	3,770	31.2
Liberia 2020	1,492	24.7	2,690	30.8
Mali 2018	1,658	35.1	4,562	21.8
Nigeria 2018	7,452	28.8	13,897	16.3
Rwanda 2020	1,322	16.3	4,844	19.2
Sierra Leone 2019	2,073	40.0	4,453	49.8
Senegal 2019	1,326	94.0	2,975	81.5
Zambia 2018	2,399	64.9	4,904	61.0
All countries	28,456	37.5	53,758	33.0

**Table 2:** Background characteristics and postnatal care service utilisation within the first 2 months after delivery among women in sub–Saharan Africa

Variables	Urban		Rural	
	Frequency	Baby Postnatal care service utilisation within 2 months after delivery (%)	Frequency	Baby Postnatal care service utilisation within 2 months after delivery (%)
***Age***	X^2^ = 8.7; p < 0.193		X^2^ = 4.4; p < 0.625	
15–19	1,708	36.9	4,232	33.3
20–24	5,596	37.9	11,321	32.8
25–29	7,704	36.8	13,008	33.0
30–34	6,306	38.3	10,539	33.0
35–39	4,605	37.9	8,583	33.5
40–44	1,883	38.1	4,371	32.0
45–49	654	33.6	1,704	31.8
***Level of education***	X^2^ = 51.9; p < 0.000		X^2^ = 246.0; p < 0.000	
No education	8,536	34.4	28,979	30.1
Primary	5,860	38.3	14,072	35.7
Secondary	11,324	39.2	9,891	37.4
Higher	2,736	38.9	816	34.6
***Wealth index***	X^2^ = 98.5; p < 0.000		X^2^ = 15.1; p < 0.005	
Poorest	1,566	25.3	18,285	33.3
Poorer	2,309	37.4	16,035	33.2
Middle	5,302	39.0	11,695	33.4
Richer	9,103	38.5	5,719	30.8
Richest	10,176	37.7	2,024	31.7
***ANC visits***	X^2^ = 95.3; p < 0.000		X^2^ = 1.4e + 03; p < 0.000	
Less than 4	8,216	33.1	24,398	24.7
4 or more	20,240	39.3	29,360	39.8
***Marital status***	X^2^ = 170.6; p < 0.000		X^2^ = 196; p < 0.000	
Never in union	2,795	36.9	3,149	34.4
Married	21,304	38.9	43,102	33.6
Cohabitation	2,670	26.3	4,989	24.7
Widowed	400	40.2	601	31.6
Divorced	522	42.9	848	41.6
Separated	765	36.0	1,069	33.7
***Frequency of listening to radio***	X^2^ = 343.4; p < 0.000		X^2^ = 895.2; p < 0.001	
Not at all	9,689	32.2	26,550	28.0
Less than once a week	7,464	40.5	11,586	36.7
At least once a week	10,783	38.9	14,921	37.1
Almost everyday	520	66.7	701	69.0
***Frequency of watching television***	X^2^ = 706.7; p < 0.000		X^2^ = 596.3; p < 0.000	
Not at all	9,436	30.3	37,525	30.1
Less than once a week	5,356	37.9	8,342	37.0
At least once a week	12,528	40.0	7,547	41.3
Almost everyday	1,136	68.6	344	64.8
***Getting medical help for self: permission to go***	X^2^ = 184.5; p < 0.000		X^2^ = 178.9; p < 0.000	
Big problem	3,813	27.6	10,302	27.4
Not a big problem	24,643	39.1	43,456	34.3
***Getting medical help for self: getting money needed for treatment***	X^2^ = 43.8; p < 0.000		X^2^ = 105.8; p < 0.001	
Big problem	11,687	35.3	29,751	31.1
Not a big problem	16,769	39.1	24,007	35.3
***Getting medical help for self: distance to facility***	X^2^ = 105.8; p < 0.000		X^2^ = 38.7; p < 0.000	
Big problem	5,764	34.0	22,901	34.4
Not a big problem	22,692	38.4	830,857	31.9

#### Binary logistic regression

In this section, we report on the determinants of women utilising PNC within the first 2 months after delivery with major emphasis on place of residence. For women in urban settings, the odds of utilising PNC service within the first 2 months after delivery was higher for women with higher education (Urban: AOR = 1.39, CI = 1.25, 1.56%). Similarly, women in rural areas in the same category of education were significantly more likely to utilize PNC within the first 2 months (AOR = 1.31, CI = 1.10, 1.58) than those with no education. Rural women in the middle (AOR = 1.15, CI = 1.08, 1.22) and richer (AOR = 1.11, CI = 1.02) wealth index were significantly more likely to access PNC within the first 2 months of delivery than those in the poorest wealth index, however, urban women in the same category showed no significant association. With respect to ANC visits, women in both urban (AOR = 1.32, CI = 1.23,1.40) and rural (AOR = 1.49, CI = 1.43,1.56) settings with 4 or more ANC had higher odds of utilising PNC within the first 2 months of delivery than those with less than 4 ANC. Compared to never married women, a significantly higher odds of PNC utilisation within the first 2 months after delivery was observed among married (AOR = 1.11, CI = 1.102,1.22) and cohabiting (AOR = 1.17, CI = 1.05, 1.31) women in rural areas but not so for urban women. The odds of urban women utilising PNC service within the first 2 months after delivery was higher for women who listen to radio at least once a week (AOR = 1.32, CI = 1.23,1.41) than those who do not listen to radio at all (see Table 3). Comparatively, similar observation was made among rural women in the same category of frequency of listening to the radio (AOR = 1.40, CI = 1.33,1.48) than those who do listen to the radio at all.

Further results show that, both urban (AOR = 0.67, CI = 0.61,0.74) and rural (AOR = 0.86, CI = 0.81, 0.91) women who had a big problem getting permission to access medical help had less likelihood of utilizing PNC within the first 2 months of delivery compared to those without such barrier in accessing PNC within the aforesaid duration after delivery. The most intriguing result in urban women is the higher likelihood of PNC uptake within the first 2 months of delivery in women who have a big problem getting money needed for treatment (AOR = 1.15, CI = 1.08, 1.23) than their counterparts without a big problem. Similar pattern of higher odds of PNC uptake was observed in rural women concerning difficulty with distance to health facility. In relation to country, urban (AOR = 0.54, CI = 0.45, 0.64) and rural (AOR = 0.89, CI = 0.80, 0.99) in Rwanda had the lowest PNC uptake within the first 2 months of delivery compared to women in Benin. However, both urban (AOR = 44.0, CI = 34.5, 56.14) and rural (AOR = 19.84, CI = 17.55, 22.42) women in Senegal utilised the highest PNC within the specified duration after delivery than those in the reference country.

**Table 3:** Binary logistic regression of PNC service utilisation within the first 2 months after delivery among women in sub–Saharan Africa

Variable	Urban	Rural
	Model 1 Adjusted Odds Ratio (95% confidence interval)	Model 2 Adjusted Odds Ratio (95% confidence interval)
***Age***		
15–19	Ref	Ref
20–24	0.99(0.88, 1.13)	0.97(0.90, 1.06)
25–29	0.96(0.85, 1.09)	1.05(0.96, 1.14)
30–34	1.02(0.91, 1.16)	1.07(0.98, 1.16)
35–39	1.06(0.80, 1.39)	1.09(0.99, 1.19)
40–44	1.08(0.93, 1.26)	1.05(0.82, 1.26)
45–49	0.93(0.75, 1.14)	1.04(0.91, 1.20)
***Level of education***		
No education	Ref	Ref
Primary	1.20***(1.10, 1.30)	1.21***(1.14, 1.28)
Secondary	1.26***(1.17, 1.36)	1.31***(1.23, 1.40)
Higher	1.39***(1.25, 1.56)	1.31***(1.10, 1.58)
***Wealth index***		
Poorest	Ref	Ref
Poorer	1.05 (0.90, 1.27)	1.09**(1.03, 1.15)
Middle	1.04(0.90, 1.24)	1.15***(1.08, 1.22)
Richer	1.00(0.87, 1.21)	1.11**(1.02, 1.20)
Richest	0.90(0.78, 1.05)	1.10(0.97, 1.25)
***ANC visits***		
Less than 4	Ref	Ref
4 or more	1.32***(1.23, 1.40)	1.49***(1.43, 1.56)
***Marital status***		
Never in union	Ref	Ref
Married	1.10(0.99, 1.21)	1.11**(1.02, 1.22)
Cohabitation	1.08(0.95, 1.23)	1.17**(1.05, 1.31)
Widowed	1.30**(1.03, 1.64)	1.10(0.89, 1.35)
Divorced	0.93(0.75, 1.19)	1.09(0.91, 1.30)
Separated	1.20*(1.01, 1.44)	1.24**(1.06, 1.47)
***Frequency of listening to radio***		
Not at all	Ref	Ref
Less than once a week	1.30***(1.21, 1.40)	1.29***(1.22, 1.36)
At least once a week	1.32***(1.23, 1.41)	1.40***(1.33, 1.48)
Almost everyday	1.14(0.92, 1.42)	1.56***(1.30, 1.86)
***Frequency of watching television***		
Not at all	Ref	Ref
Less than once a week	1.16***(1.06, 1.26)	1.15***(1.08, 1.22)
At least once a week	1.11**(1.03, 1.21)	1.15***(1.07, 1.24)
Almost everyday	1.34**(1.12, 1.61)	0.96(0.76, 1.30)
***Getting medical help for self: permission to go***		
Big problem	0.67***(0.61, 0.74)	0.86***(0.81, 0.91)
Not a big problem	Ref	Ref
***Getting medical help for self: getting money needed for treatment***		
Big problem	1.15***(1.08, 1.23)	0.97(0.92, 1.02)
Not a big problem	Ref	Ref
***Getting medical help for self: distance to facility***		
Big problem	1.02(0.95, 1.10)	1.13***(1.07, 1.18)
Not a big problem	Ref	Ref
**Country**		
Benin 2018	Ref	Ref
Cameroon 2018	1.27***(1.12, 1.43)	1.50***(1.34, 1.67)
Gambia 2020	3.05***(2.70, 3.43)	8.60***(7.67, 9.63)
Guinea 2018	2.02***(1.76, 2.31)	2.43***(2.19, 2.69)
Liberia 2020	0.93(0.80, 1.08)	1.79***(1.60, 2.01)
Mali 2018	1.75***(1.53, 199)	1.30***(1.17, 1.44)
Nigeria 2018	1.07(0.96, 1.18)	0.89***(0.81, 0.97)
Rwanda 2020	0.54**(0.45, 0.64)	0.89***(0.80, 0.99)
Sierra Leone 2019	1.97***(1.74, 2.23)	4.39***(3.97, 4.84)
Senegal 2019	44.0***(34.5, 56.14)	19.84***(17.55, 22.42)
Zambia 2018	5.30***(4.55, 6.17)	6.55***(5.89, 7.29)

*p < 0.05 ** p < 0.01 ***p < 0.001 ref = reference category

## Discussion

In the present study, we examined the utilisation of PNC services within the first 2 months after childbirth in SSA. Our findings indicate that PNC prevalence was 37.5% and 33% in the urban and rural residences respectively. The higher proportion of PNC service utilisation within the first two months among women in the urban could be explained from the perspective that women in this type of residency often have many contextual advantages that promote better health seeking behaviour as compared to rural residences [24]. For instance, a study conducted in Ghana [25] revealed that compared to urban dwelling women, those in rural residences had to cover more than 4 km to reach the nearest health facility, and each kilometer significantly reduced the likelihood to utilise PNC. Additionally, the study showed that there were country differences in the likelihood of utilising early PNC services within the first two months. In both rural and urban areas, Senegal reported the highest odds of early PNC service utilisation within the first two months whereas Rwanda reported the least likelihood. The country differences could be as a result of the different socio-cultural contexts, and the availability of maternal healthcare policies.

Our study revealed that in both urban and rural residences, women with increasing level of formal education was associated with higher likelihood to utilise PNC services within the first two months. This finding is consistent with previous studies conducted in Nigeria [26] and Ethiopia [27, 28] that have reported that having higher level of formal education is associated with higher likelihood of PNC utilisation within the first 2 months after delivery. This could be because women with formal education tend to be empowered and are more likely to be aware of the benefits of seeking PNC services [26]. Hence, informing their decisions to utilise PNC services within the first two months. It is also possible that women who have higher level of formal education may be in higher socio-economic status that allows them to easily afford the expenditure that characteristics the utilisation of maternal healthcare services including PNC within the first two months.

The present study also found that having four or more ANC contacts was associated with a higher probability to utilise PNC services within the first two months, in both urban and rural areas. Similar findings have been reported in Abebo and Tesfaye [29] who reported that Southern Ethiopian women who had four or ANC contacts were 9.5 times more likely to utilise PNC services within the first two months. Our result is further corroborated by a previous study conducted in Nepal [30]. It is possible to explain this observation from the point that ANC visits offer an opportunity for women to have access to counselling and information about what to expect and do after childbirth – PNC attendance is among the activities that women are encouraged to undertake during the health promotion and counselling session at ANC clinics. Another perspective to this finding could be the high level of trust and confidence in the healthcare system that comes as a result of regular ANC visits [31]. It is also possible that those with an easier access to ANC services were the same women who were able to utilize PNC within the first 2 months after delivery. Thus, suggesting that accessibility could be a latent possible explanation for the observed association.

Exposure to the media (i.e., radio and television) was also a significant predictor of PNC service utilisation within the first two months, in both urban and rural residencies. The result is in alignment with Debie and Tesema’s study [32] that found media exposure to be associated with 1.42 times more likelihood of utilising PNC services within the first two months. A plausible explanation for the observed association could be exposure to media brings about a significant improvement in women’s awareness and the need to consider PNC uptake within the first two months, even if they expect to have a home-based delivery [33]. The study also showed that compared to women who had never been in union those who were married were more likely to utilise early PNC services. Perhaps, those in union would be supported by their partners (usually financially) to utilise PNC services within the first two months [33].

The study also revealed that having problem with distance was significantly associated with lower odds of utilising early PNC services among women in rural areas but not in the urban areas. In most countries in SSA, the healthcare architecture and system is centralized in the urban areas, thereby causing disparate distribution of healthcare facilities [15]. Hence, the nearest healthcare facility in the rural areas is usually several miles away [16]. On the other hand, having problem with getting money needed for treatment was significantly associated with higher odds of having PNC utilisation among women in the urban areas, not the rural areas. It is unclear the reasons for this difference. Perhaps, a qualitative study would be needed in the future to unearth how and why having problem with getting money needed for treatment was only significant in predicting PNC utilisation within the first two months among women in the urban areas but not in the rural areas.

Requiring permission to go to the health facility emerged as a significantly associated with lower likelihoods of utilising PNC services within the first two months among women in both urban and rural areas. This result is in consensus with prior evidence that shows that women’s autonomy is significantly associated with the utilisation of maternal healthcare services including PNC utilisation within the first two months [14, 17]. A possible explanation for this finding could be that, women whose partners refuse to grant them the permission to go to the healthcare facility would be unable utilise PNC services within the first two months. This presents a significant challenge irrespective of the place of residence.

### Policy implication

Based on the finding from this study, it is imperative for the governments of the SSA countries to improve ANC attendance in both rural and urban areas. This would require health providers to leverage ANC sessions as a conduit to counsel women and educate them about the need for them to initiate PNC early. The findings also underscore a need for maternal healthcare services to decentralised in the rural areas to reduce the burden that distance poses in the utilisation of PNC services. Given that media exposure was an enabling factor, it must be harnessed and utilized as a tool to raise awareness and empower women to seek an early utilisation of PNC services. Our findings underscore the point that PNC is not free in every included country and therefore that could be an unstudied barrier its utilisation within the first 2 months after delivery.

### Strength and limitations

The strength of this study lies in the use of large dataset that has been validated. This allows us to generalise the findings to women across the eleven countries included in this study. A major challenge with the use of DHS data is that, it precludes the establishment of causal inferences due to its reliance on cross-sectional study design. We are limited in the kind of inferences that can be made, as we are only able to make associations between the factors identified and the PNC service utilisation. Also, questions about PNC were self-reported. Hence, there is the possibility of recall and social desirability bias which is beyond the control of the authors. Another limitation is that the DHS has no standardised definition for urban and rural which makes it difficult to find a fitting definition.

## Conclusion

In this study, we conclude that the PNC service utilisation within the first 2 months after delivery was low across rural and urban residences. Level of education, ANC visits, media exposure, requiring permission to seek healthcare, and marital status were significantly associated with PNC service utilisation within the first 2 months after childbirth, in both rural and urban areas. However, wealth status and having a problem with distance were significant in only rural areas. There is, therefore, a need for SSA countries to develop population tailored interventions such as advocacy and health education targeted at women with no formal education in both rural and urban areas. Our study also suggests that SSA countries must intensify radio programs and advertisements on the health benefits of seeking PNC within the first 2 months after delivery to improve maternal and child health. Also, pro-poor services would be needed in rural areas to create an enabling environment that would support the utilisation of PNC services. There is a need to increase the availability and accessibility to community and primary healthcare facilities in rural areas in SSA. We conclude that the reasons why having a problem with money for treatment was significant in urban areas but not in rural areas may be complex and likely depend on various contextual factors, including differences in economic resources, healthcare infrastructure, and social support systems. Hence, underscoring a need for policy makers to focus on initiatives that enhance economic resources, healthcare infrastructure, and social support systems, especially those from low-income households, to improve their ability to afford PNC services. Given the significant differences in PNC service utilisation across the countries, it is imperative for each SSA country included in this study to develop specific interventions, programmes and policies that meets their context-specific needs.

## Electronic supplementary material

Below is the link to the electronic supplementary material.

Supplementary Material 1

## Data Availability

Data were available in a public via the measuredhs website at https://dhsprogram.com/data/available-datasets.cfm.

## Abbreviations

ANC: Antenatal care
DHS: Demographic and health survey
PNC: Postnatal care
SSA: Sub-Saharan Africa
WHO: World Health Organization
